# A disease potential-driven graph attention model for comorbidity risk prediction of hypertension

**DOI:** 10.3389/fdata.2026.1814157

**Published:** 2026-04-02

**Authors:** Leming Zhou, Hanshu Qin, Yanmei Yang, Gang Huang, Zhigang Liu

**Affiliations:** 1 School of Computer Science and Technology, Chongqing University of Posts and Telecommunications, Chongqing, China; 2 The First Affiliated Hospital of Chongqing Medical University, Chongqing, China; 3 Chongqing University of Traditional Chinese Medicine, Chongqing, China; 4 Department of Cardiology, The Third People's Hospital of Chengdu, Chengdu, China; 5 School of Computer Science and Technology, Dongguan University of Technology, Dongguan, China

**Keywords:** comorbidity network, disease potential, fusion attention, hypertension, risk prediction

## Abstract

Hypertension is associated with an increased risk of serious complications, and the hazards are very serious. However, current methods for predicting comorbidity risks face the challenge that comorbidity prediction relying solely on data driven may lead to clinically implausible associations and reduce model interpretability. Also, how to capture the fusion features of patient and identify differences among them to facilitate risk prediction needs to be addressed. To overcome these challenges, we propose a Disease Potential-Driven Graph Attention (DP-GA) model for comorbidity risk prediction of hypertension, which has 3-fold ideas: (a) Constructing a fusion mechanism for the correlation among the patients' disease features and the structural, thus integrating feature attention and structural attention effectively; (b) Introducing a similarity-difference balance mechanism to further identify the relationships among patients; and (c) Designing a disease potential-driven attention mechanism to calculate the disease potential and construct masks, thus preserving the effective associations from high-risk patients to low-risk patients. Experimental results demonstrate that our proposed DP-GA model achieves a significant improvement in comorbidity risk prediction for patients with hypertension across three comorbidity datasets collected by the research group, compared with both the baseline and state-of-the-art peer methods. We also analyze the comorbidity network to predict the risk of hypertension comorbidity, thereby improving interpretability and early prediction of such comorbidities.

## 1 Introduction

The World Health Organization (WHO) reports that approximately 1.1 billion adults worldwide are affected by hypertension. Commonly, patients with hypertension often have various comorbidities and complications, e.g., chronic obstructive pulmonary disease (COPD), diabetes mellitus (DM), and coronary heart disease (CHD), which pose significant risks. According to the 2019 Global Burden of Disease Study, the total number of COPD cases was 212.3 million, with 16.2 million new COPD cases reported annually. COPD becomes the third leading cause of death globally, after ischemic heart disease and stroke, with an estimated 3.324 million deaths. COPD not only affects the lungs but also often coexists with other systemic diseases. The comorbidities are not only related to the acute exacerbation of COPD (AECOPD) but also have a heavy medical and economic burden. Heubel et al. (2021) have found that AECOPD is associated with an increased risk of cardiovascular events and may be related to endothelial dysfunction. Common comorbidities related to COPD include cardiovascular disease (CVD), lung cancer, asthma, diabetes, metabolic syndrome, depression, chronic kidney disease, gastrointestinal disease, and anemia, etc. (Gong et al., 2024; Xu and Yew, 2023; Zhang and Tong, 2025; Lu et al., 2021; Goh and Hartman, 2025).

Although hypertension, CHD, DM, and COPD all have relatively mature and systematic diagnostic and treatment strategies, when the two coexist, the different specialties of first-visit physicians can easily lead to missed or misdiagnoses, resulting in delayed treatment. Therefore, clarifying the above diagnoses of comorbidities, early screening and diagnosis, and concurrent treatment of comorbidities are key to diagnosing and treating patients with comorbidities. However, the challenge of hypertension comorbidity lies in the synergistic effects among different diseases, which overlap or mask symptoms, resulting in ambiguous diagnostic clues, data presenting characteristics of high-dimensional sparsity and non-linearity, complicating the prediction of comorbidity risks, fragmenting management, and making it difficult to distinguish different complications, which are highly similar in manifestation and hard to differentiate (Bao et al., 2023; Abess et al., 2023; Wang et al., 2023; Arakelyan et al., 2023; Ni et al., 2023; Wu et al., 2026; He et al., 2025). This makes it difficult to identify comorbidities, increasing the risk of misdiagnosis and missed diagnoses. It not only increases the risks for patients but also exacerbates the contradictions in doctor-patient relationships and the allocation of medical resources. Moreover, a data-driven approach based solely on deep learning makes it difficult for doctors to trust and adopt the prediction results. These challenges urgently require interdisciplinary research in medicine and artificial intelligence to address them and have become a research hotspot in the field of hypertension comorbidity prediction.

Some studies on post-classification prediction, represented by comorbidity graph neural networks have been conducted (Tian et al., 2025; Che and Wang, 2025). For instance, Abuhantash et al. (2024) propose a prediction method for Alzheimer's disease based on graph neural networks and achieved good results. Dong et al. (2022) propose a graph convolutional network, i.e., MorbidGCN, to predict the coexistence of multiple diseases by integrating population phenotypes and disease networks. Combining disease comorbidities to extract drug and disease characteristics can reduce the time and cost of developing new drugs (Luo et al., 2024). The adoption of a pre-embedding learning method based on hypergraphs is helpful for predicting new associations between comorbidity pairs (Biswas et al., 2025). Moreover, to address interpretability issues, causal graph learning-based methods have emerged. For instance, a causal graph learning method based on information-bottleneck constraints is useful for denoising (Yuan et al., 2024). Counterfactual interpretation methods based on causal intervention can reduce false correlations (Shao et al., 2024; Våle et al., 2025). The graph attention encoder method based on causal discovery has been shown to be effective at solving hypothesis-violation problems (Liu et al., 2024).

Some scholars have also studied causal inference in the context of comorbidities. For instance, Li et al. (2025) explore the causal associations between 35 modifiable factors and cardiovascular metabolic polypathy, as well as each individual disease. Zeng et al. (2025) quantify the prevalence of depression among patients with cardiovascular diseases and its impact on mortality through meta-analysis, verifying the causal relationship between cardiovascular diseases and depression. Chen et al. (2023) analyze the association between comorbidity status and the development trajectory of comorbidity and dementia in the elderly population.

Although researchers have provided feasible methods for predicting these severe comorbidities (Fu et al., 2024; Glyde et al., 2024; Yin et al., 2024; Bonomo et al., 2022; Yang et al., 2024; Kang et al., 2024; Pikin et al., 2024; Heid and Green, 2022), the current methods for predicting comorbidity risks encounter the following difficulties: Firstly, due to the complexity of the association of hypertension comorbidities, there are non-linear and multi-factor interaction patterns in the association with other concurrent and comorbid diseases such as DM, CHD, and COPD, making it difficult to capture the characteristics and relationships of patients using traditional methods. Secondly, the traditional attention mechanism often focuses on similar nodes, leading to convergence of learned node representations and loss of individual node characteristics. Accurately modeling these individual differences is a key challenge in personalized prediction. Thirdly, the traditional approach neglects the relationships among disease risks and lacks the logical connections among diseases in clinical practice.

This study aims to identify potential diseases and mechanisms related to the development of comorbidities of hypertension with these severe comorbidities. By analyzing the comorbidity networks of COPD, DM, and CHD, the study focuses on the high-incidence diseases in the case group and their pathological mechanisms. Explore comorbidity intervention strategies based on a common pathological mechanism. To this end, the paper proposes a Disease Potential-Driven Graph Attention (DP-GA) model for hypertension comorbidity risk prediction. The modeling framework is built upon three core mechanisms: (a) integrating feature attention with structural attention to jointly capture feature correlations and graph topology; (b) modeling patient relationships through a similarity-difference balance mechanism that accounts for both similarity and distinction; and (c) estimating disease potentials and constructing masks to preserve clinically meaningful directional associations from high-risk to low-risk patients. These mechanisms collectively enhance representation learning and aware prediction for comorbidity risks.

In general, the paper aims to make the following main contributions:

- A unified fusion mechanism which integrates patient's disease features with network structure information. By introducing latent position embeddings to capture network topology and computing structural attention, the model achieves dual fusion of feature and structural attention with adaptive weighting, providing a more comprehensive informational basis for comorbidity risk prediction.
- Establish a similarity-difference balance mechanism that models complicated patient relationships. Using Bregman divergence as a difference metric, the mechanism jointly considers similarity and distinction between nodes, avoiding representation homogenization while preserving node-specific attributes through adaptively balanced difference information.
- A disease potential-driven attention network that incorporates clinical disease correlation logic. By calculating disease risk potential from patient characteristics and constructing masks based on it, the method simulates directional disease influence and imposes constraints on attention weights, thereby enhancing interpretability and reflecting realistic risk propagation pathways.

Experimental evaluation on three hypertension comorbidity datasets shows that the proposed DP-GA model consistently outperforms existing baseline and state-of-the-art methods in predicting comorbidity risks, demonstrating that our method can identify patients at high risk of hypertension-related complications in advance. These results highlight the model's capability to support early risk identification and clinically interpretable prediction in comorbidity management.

The reminder of this paper is organized as follows. Section 2 states the data material and problem statements. Section 3 presents the DP-GA model. The experimental results are discussed in Section 4. Finally, Section 5 concludes this paper.

## 2 Data material and problem statements

### 2.1 Dataset preparation

This retrospective study is based on the electronic medical record (EMR) homepage of anonymous discharged patients from a tertiary hospital from 2019 to 2023. The inclusion and exclusion labels are based on guidelines and indicators. The study was approved for hospital scientific review and passed the ethics review. With ICD-10 disease coding classification based on relevant clinical guidelines and other related literature and expert consultation, we have developed the following inclusion and exclusion criteria: due to our research objective of hypertension combined with COPD, DM and CHD, we excluded the acute infectious disease categories in sections A and B in ICD-10 coding, as well as the tumor diseases in section C coding, to achieve preliminary screening. To demonstrate the effectiveness of our proposed method, three groups of hypertension comorbidities datasets have been designed based on inclusion and exclusion criteria. The case group is defined as a total of hospitalized patients diagnosed with hypertension, and before suffering from chronic obstructive pulmonary disease (COPD, J44), diabetes mellitus (DM) and coronary heart disease (CHD), while the control group consists of patients with only primary hypertension and other complications, excluding COPD, DM and CHD. As can be seen from Table 1, the three datasets, i.e., COPD, DM and CHD, have 630, 1,024, and 1,668 nodes, respectively, and the features all reach hundreds of dimensions. The datasets are binary datasets, with the control group and the case group each accounting for half.

**Table 1:** Basic information of the case group and control group datasets.

Category	COPD numbers	Disease feature	DM numbers	Disease feature	CHD numbers	Disease feature
Control group	315	355	512	435	834	500
Case group	315	355	512	435	834	500
Group	630	355	1,024	435	1,668	500

### 2.2 Problem statements

Assume that the patient interaction network is denoted by *G*_*p*_={*V, E*, X}, where *G*_*p*_ denotes the patient graph, *V*={*v*_1_, *v*_2_, …, *v*_*Z*_} is the node set of *Z* patient nodes, *E* denotes the patient edges (connections) based on the same disease, |*E*| represents the number of connections between the patients, XεR^Z × *F*^ represents the input node feature matrix of patients, and *F* represents the dimension of features of each patient node. The adjacency matrix of *G*_*p*_ is denoted by **D**ε{0, 1}^Z × *Z*^. In the following, we use **W** to denote the weight matrix, **H** to denote the feature matrix of patient nodes. In this work, we adopt classification to predict the probability that a patient has a specific disease, such as DM, CHD, or COPD. *C*_*L*_ denotes the number of categories in the classification, and the prediction of hypertension comorbidity refers to the predicted outcome of the disease based on the learned patient features. We denote the set consisting of their directly adjacent nodes as N(i). When a patient's outcome is diagnosed as the target disease, the label value is 1; otherwise, it is 0.

## 3 Methods

### 3.1 Overview of the proposed DP-GA model

**Figure 1:**
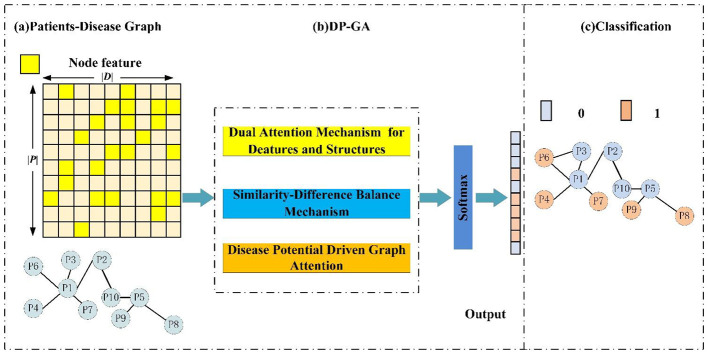
The framework of the proposed DP-GA model. **(a)** Patient-Disease graph. **(b)** DP-GA. **(c)** Classification.

DP-GA is a graph neural network framework specifically designed for predicting comorbidity, and it has constructed an end-to-end process for comorbidity risk prediction. In the process of comorbidity risk prediction, the first step is to construct a dual attention mechanism combining feature attention and structural attention to calculate the basic attention. At the same time, it integrates node position embedding information to capture the positional information between patients, and through latent position learning, it captures the positional changes of patient nodes in the latent space. The second step involves the application of Bregman divergence to capture the differences between nodes, and the calculation of the difference metric is utilized in the subsequent adjustment of attention weights to implement the mechanism of similar-differentiated information transmission. The third step involves introducing the disease correlation logic from clinical practice and proposing a disease-potential-driven attention mechanism. By using masking to simulate the reasonable direction of disease transmission, this mechanism imposes constraints on the previously obtained attention weights, thereby effectively enhancing the accuracy and interpretability of comorbidity prediction. The DP-GA framework is illustrated in Figure 1.

### 3.2 Fusion attention mechanism

To overcome the limitations of information utilization, a dual-attention mechanism combining feature and structural attention was developed (He et al., 2021, 2024). At the same time, the similarity of patients' features and their structures was calculated to more accurately measure the influence among patients.

Generally, we calculate the attention score between two patients *i* and *j* as follows:


eijbase=ai⊤LeakyReLU(Whi)+aj⊤LeakyReLU(Whj), (1)

where $e_{ij}^{base}$ denotes the patient's base attention, capturing the explicit risk factors of historical diseases. *a*_*i*_ and *a*_*j*_ denote the attention distributions for the disease features of patients *i* and *j*, respectively, *h*_*i*_ and *h*_*j*_ are the original disease feature vectors of patients *i* and *j* in Equation 1. **W** is a weight matrix that maps patients' high-dimensional disease features.

However, the attention score defined above relies solely on transformed node features, without incorporating the topological positions of the nodes within the graph. To construct a more comprehensive attention mechanism, it is necessary to integrate structural information into the base attention formulation. To address the underutilization of topological information in comorbidity prediction, we learn node position representations in a latent space, thereby uncovering the underlying structural relationships among patient nodes. This allows the model to leverage both feature-based and structure-based information for more accurate comorbidity inference.

To capture positional relationships among patient nodes, we further introduce a structural attention mechanism that models implicit structural associations among patients. The structural attention score is calculated as follows:


$$\begin{array}{c} e_{ij}^{pos}={\left(s_{i}⊙a_{s}\right)}^{⊤}\left(s_{j}⊙a_{s}\right), \end{array}$$ (2)

where $e_{ij}^{pos}$ denotes the structural attention score between patients *i* and *j*, serving as a quantitative measure of the structural similarity between the two nodes, *a*_*s*_ is the positional attention vector, *s*_*i*_ and *s*_*j*_ denote the structural feature vectors of the respective patient nodes, which are derived by learning latent position embeddings that encode the topological structure of the patient network in Equation 2.

To enable the model to jointly leverage both nodal feature information and graph structural information, we integrate the learned positional representations into the attention mechanism. This enhances the model's capacity to reason about comorbidities by combining explicit feature affinities with implicit structural proximities. The final integrated attention score in Equation 3 is obtained by fusing the feature-based and structure-based components:


$$\begin{array}{c} e_{ij}^{fusion}=e_{ij}^{base}+e_{ij}^{pos}, \end{array}$$ (3)

### 3.3 Difference perception mechanism

To overcome the limitations of conventional attention mechanisms, which primarily focus on node similarity, we propose a dual-perspective framework that models both node similarities and differences. This approach enables more effective information propagation and enhances graph representation learning by adaptively strengthening or attenuating messages based on node relations.

#### 3.3.1 Bregman divergence for node difference

To quantify the dissimilarity between patients in both feature and structural spaces, we employ Bregman divergence as a principled measure of difference (He et al., 2021, 2024). This allows the model to support personalized comorbidity prediction by explicitly accounting for patient heterogeneity. The composite Bregman divergence between node and node is defined as


$$\begin{array}{c} d_{ij}=\omega \times d_{ij}^{\left(f\right)}+\left(1-\omega \right)\times d_{ij}^{\left(s\right)}. \end{array}$$ (4)

where *d*_*ij*_ denotes the integrated difference measure from node *i* to node *j* in Equation 4. $d_{ij}^{\left(f\right)}$ and $d_{ij}^{\left(s\right)}$ represent the Bregman divergence in the feature space and structural space, respectively, capturing the respective discrepancies. ωε[0,1] is a trainable weighting factor that balances the contributions of feature-level and structure-level differences.

#### 3.3.2 Similarity-difference guided message passing

The Bregman divergence provides a differentiable difference metric that is subsequently used to modulate attention weights (He et al., 2021, 2024). This leads to a similarity-difference aware message-passing scheme, where propagation is enhanced between similar nodes and suppressed between dissimilar ones. Let the node features and position embeddings at layer be transformed as:


$$\begin{array}{c} H^{\left(l\right)}=H^{\left(l\right)}W_{h}^{\left(l\right)},P^{\left(l\right)}=P^{\left(l\right)}W_{p}^{\left(l\right)}. \end{array}$$ (5)

**H**^(*l*)^ denote the feature matrix of patient nodes, **P**^(*l*)^ denote the latent positions, **W**^(*l*)^ to denote the weight matrix in Equation 5. We then construct a joint relation matrix **R** that incorporates both similarity and difference:


$$\begin{array}{c} R_{ij}=D_{ij}\cdot \exp\left(\frac{C_{ij}-\beta O_{ij}}{\tau }\right), \end{array}$$ (6)

where **C** and **O** are learnable matrices that compute feature-structure similarity and difference, respectively; β is a scaling coefficient, and τ is a temperature hyperparameter in Equation 6. **D**_*ij*_ denotes the adjacency matrix of *G*_*p*_.

Messages are aggregated as:


$$\begin{array}{c} M_{i}=\underset{jN\in \left(i\right)}{\sum }\frac{R_{ij}}{\sqrt{\sum_{k\in N\left(i\right)}R_{ik}}}\cdot H_{j}, \end{array}$$ (7)

By the combination of the joint effect of **C** and **O**, a matrix M is constructed in Equation 7.

Finally, the updated node representation combines self-information and aggregated messages:


$$\begin{array}{c} H_{i}^{\left(l+1\right)}=\mu \left(\frac{1}{N\left(i\right)}H_{i}\right)+M_{i}, \end{array}$$ (8)

where μ is a learnable positive irrational, **H**_*i*_ represents the feature vector of node *i* in Equation 8. The resulting propagation mechanism strengthens information flow between similar nodes while reducing influence from dissimilar nodes. The attention score is accordingly adjusted by the difference measure:


$$\begin{array}{c} e_{ij}^{adjust}=e_{ij}^{fusion}-\delta \cdot d_{ij}, \end{array}$$ (9)

where δ is a learnable scaling parameter. The final attention weight is obtained by normalizing $e_{ij}^{adjust}$ over all neighbors in Equation 9.

This unified similarity-difference framework enables the model to dynamically balance homophily and heterophily in the patient graph, leading to more expressive and discriminative representations for comorbidity prediction.

### 3.4 Disease potential-driven attention

While the previously described modules are primarily data-driven, relying solely on statistical correlations may lead to clinically implausible associations and reduce model interpretability. To ground the model in established clinical knowledge, we introduce the disease correlation logic in clinical practice, designed to quantify the influence intensity of disease status by calculating the disease potential of patient nodes, and propose a disease potential-driven attention mechanism. This component calculates the disease potential based on the patient's disease-related features, and then constructs masks based on the disease potential to impose constraints on the attention weights, thereby learning node representations that conform to disease patterns.

#### 3.4.1 Disease potential calculation

To assess the severity or progression risk of a target disease for each patient, we compute a disease potential score based on clinically relevant nodal features. This potential represents the propensity or risk level of a patient node for the disease under consideration. The potential for patient is obtained by calculating the difference in potential between the source node and the target node, and a mask based on the direction of disease transmission is created to influence the calculation of attention weights, i.e.,


$$\begin{array}{c} eq10p_{i}=\sigma \left(\sum_{q\in D}X_{iq}\right). \end{array}$$ (10)

*p*_*i*_ represents the risk or probability of the target disease occurring in patient *i, q* is an index of disease-related features. *X*_*iq*_ represents the value of the *i*-th sample at the *q*-th feature, σ(·) is the sigmoid function, which converts the linear combination result into a probability value in Equation 10. D is the set of disease features.

#### 3.4.2 Mask construction

In order to restrict the direction of information flow and only allow nodes with high disease potential to transfer information to nodes with low disease potential, we construct a binary causal mask. This mask permits attention flow only from nodes with strictly higher disease potential to those with lower or equal potential, thereby encoding an asymmetric, risk-informed constraint. For a directed edge from source node *i* to target node *j*, the mask coefficient is defined as:


$$m_{ij}=\{\begin{array}{cc} 1 & if\;p_{i}>p_{j};\\ \epsilon & otherwise. \end{array}$$ (11)

Here, ϵ is a small positive constant (e.g., 0.1) applied to connections that do not satisfy the direction in Equation 11, effectively suppressing—rather than completely eliminating—the information flow. This ensures numerical stability while strongly biasing the model toward clinically plausible pathways.

#### 3.4.3 Integration with attention mechanism

The mask is integrated into the attention mechanism via element-wise multiplication with the unconstrained attention weights. This constrained attention weights that align with disease progression logic, i.e.,


$$\begin{array}{c} \alpha_{ij}^{potential}=\frac{\exp\left(e_{ij}^{adjust}\right)\cdot m_{ij}}{\sum_{k\in N\left(i\right)}\exp\left(e_{ik}^{adjust}\right)}, \end{array}$$ (12)

where $e_{ij}^{adjust}$ is the adjusted attention score from Equation 9. We denote the set consisting of their directly adjacent nodes as N(i) in Equation 12. The resulting weights are then used to perform message passing and feature aggregation in Equation 13, i.e.,


$$\begin{array}{c} h_{i}^{\left(l+1\right)}=\mu \left(\sum_{j\in N\left(i\right)}\alpha_{ij}^{potential}\cdot Wh_{j}^{\left(l\right)}\right). \end{array}$$ (13)

This design ensures that the node representations are updated primarily along directions consistent with clinical risk gradients, thereby enhancing the model's clinical validity and robustness against spurious correlations.

### 3.5 Loss function

To train the proposed model end-to-end, we formulate a composite loss function that jointly optimizes the accuracy of comorbidity prediction and the preservation of inherent patient graph structure. This ensures the model not only performs accurate classification but also learns representations that are topologically meaningful.

#### 3.5.1 Classification loss

The aggregated node representations, refined through the proposed attention mechanisms, are passed through a final classification layer, e.g., a fully connected network with Softmax activation, to generate the predicted probability ŷ_*i*_ of comorbidity for patient *i* in Equation 14. The primary objective is optimized using the binary cross-entropy loss, which measures the discrepancy between predictions and ground-truth labels:


$$\begin{array}{c} L_{c}=-\frac{1}{Z}\sum_{i=1}^{Z}\left[y_{i}\log\left({ŷ}_{i}\right)+\left(1\;-y_{i}\right)\log\left(1-{ŷ}_{i}\right)\right], \end{array}$$ (14)

where *Z* is the total number of patient samples, *y*_*i*_∈{0, 1} is the ground-truth comorbidity label, and ŷ_*i*_∈(0, 1) is the predicted probability. This loss drives the model to correctly identify patients at risk of comorbidities.

#### 3.5.2 Position preservation loss

To ensure that the learned latent position embeddings *h*^*p*^ faithfully reflect the topological structure of the patient graph (He et al., 2021, 2024), we introduce a position preservation loss. This is implemented as a graph Laplacian regularization term, which constrains neighboring nodes in the graph to have similar position embeddings in the latent space. The loss is defined as:


$$\begin{array}{c} L_{p}=\sum_{\left(i,j\right)\in \varepsilon }w_{ij}||h_{i}^{p}-h_{j}^{p}|{|}_{2}^{2}, \end{array}$$ (15)

where $h_{i}^{p}$ and $h_{j}^{p}$ are the position embeddings of nodes *i* and *j*, E is the set of edges in the graph, *w*_*ij*_ is the weight of the edge connecting them in Equation 15. Minimizing *L*_*p*_ enforces smoothness in the embedding space with respect to the original graph connectivity, thereby explicitly preserving structural information that is crucial for robust relational inference.

#### 3.5.3 Total loss

The final training objective is a weighted sum of the classification loss and the position preservation loss in Equation 16, i.e.,


$$\begin{array}{c} L_{total}=L_{c}+{\lambda L}_{p}. \end{array}$$ (16)

The hyperparameter λ ≥ 0 controls the trade-off between predictive accuracy and structural fidelity. By jointly optimizing *L*_*total*_, the model learns representations that are simultaneously discriminative for the downstream prediction task and structurally coherent, enhancing both performance and interpretability in the clinical application context.

### 3.6 Comorbidity networks analysis

To explain the reasons for the differences in the risk prediction of comorbidity among patients from the perspective of comorbidity, we also analyzed the correlation of comorbidity networks. It can be expressed as follows:


$$\begin{array}{c} \varphi_{uv}=\frac{N_{uv}Z-Y_{u}Y_{v}}{\sqrt{Y_{u}Y_{v}\left(Z-Y_{u}\right)\left(Z-Y_{v}\right)}}. \end{array}$$ (17)

where *N*_*uv*_ is the number of patients affected by the same diseases, *Z* is the total number of patients, *Y*_*u*_ and *Y*_*v*_ are the prevalences of diseases *u* and *v* in Equation 17, respectively (Hidalgo et al., 2009).

## 4 Experiments

### 4.1 General settings

We divided the dataset into training set, validation set and test set in the ratio of 6:2:2. The hyperparameters λ, η, *hid, drop*, and *heads* represent the regularization coefficient, learning rate, hidden layer dimension, dropout rate, and the number of heads, respectively, β and μ are the learnable parameters of the model, and no manual parameter tuning is required. The specific settings are as follows:

On the COPD dataset, the values of λ, η, *hid, drop*, and *heads* are 1, 0.0005, 64, 0.3, and 4, respectively. On the DM dataset, the values of λ, η, *hid, drop*, and *heads* are 1, 0.0005, 64, 0.3, and 4. On the CHD dataset, the values of λ, η, *hid, drop*, and *heads* are 1, 0.001, 64, 0.3, and 4. For each baseline model in the three datasets, we conducted five runs to obtain the average performance, with each run lasting for 1,000 rounds. The experiments have been conducted on a PC with a 3.20 GHz i9 CPU and 32 GB RAM. All the models are implemented in Anaconda 3.8. To demonstrate the effectiveness of our proposed DF-GA model, three groups of hypertension comorbidities datasets have been designed based on inclusion and exclusion criteria, as summarized in Table 1. Their details have been provided in Section 2.1.

We adopt several baselines and state-of-the-art GNN models, including APPNP (Klicpera et al., 2019), DGI (Veličković et al., 2019), GCN (Kipf and Welling, 2017), JKNET (Xu et al., 2018), SGC (Wu et al., 2019), and GraphSAGE (Hamilton et al., 2017) as peer methods to evaluate the effectiveness of the proposed DP-GA model by comparing it with them for comorbidity risk prediction.

### 4.2 Evaluation metrics

To evaluate the classification prediction performance of the model, both accuracy (ACC) and F1 score (F1) are used to evaluate the co-morbidity prediction performance. ACC is a commonly used metric for evaluating classification predictions, representing the proportion of correctly classified samples out of the total sample count. The formula is as follows:


$$\begin{array}{c} ACC=\frac{XP+XN}{XP+XN+EP+EN}. \end{array}$$ (18)

Equation 18 refers to the proportion of correctly predicted samples among all samples. Here, *XP* represents true positive samples, *XN* represents true negative samples, *EP* represents false positive samples, and *EN* represents false negative samples.

Since our model is designed for classifying and predicting patient comorbidities, the F1 evaluation metric is also suitable for the patient classification task. Therefore, we employed the F1 metric simultaneously as follows:


F1=2Pre×RecPre+Rec. (19)

*Pre* represents precision, *Rec* represents recall rate in Equation 19. First, we calculate the average of precision and recall rate, and then use the formula to obtain F1.

### 4.3 Comparison results and analysis

To evaluate the efficacy of the proposed method, we conducted a series of comparative experiments. A patient comorbidity network was constructed based on disease co-occurrence relationships. As shown in Table 1, the historical disease vectors of patients prior to the onset of COPD, DM, and CHD were utilized as node features. The patient nodes were classified using our DP-GA model alongside six representative graph neural network baseline models. Experimental results are summarized in Table 2. The results demonstrate that our DP-GA model achieves superior performance across all evaluation metrics. This improvement can be attributed to the model's ability to effectively integrate nodal features, topological structure, and clinically-informed constraints, thereby learning more discriminative and robust node embeddings from the graph-structured data.

**Table 2:** Performance comparison results of our proposed DP-GA model and the compared GNN models across ACC and F1 metrics.

Methods	COPD		DM		CHD	
	ACC	F1	ACC	F1	ACC	F1
DP-GA (our)	0.8873 ±0.0154	0.8872 ±0.0155	0.7210 ±0.0078	0.7165 ±0.0053	0.7976 ±0.0084	0.7975 ±0.0083
APPNP	0.8683 ± 0.0268	0.8680 ± 0.0266	0.6864 ± 0.0376	0.6766 ± 0.0366	0.7546 ± 0.0330	0.7519 ± 0.0346
DGI	0.8302 ± 0.0357	0.8294 ± 0.0356	0.6874 ± 0.0270	0.6871 ± 0.0271	0.7534 ± 0.0254	0.7527 ± 0.0250
GCN	0.8397 ± 0.0380	0.8379 ± 0.0385	0.6981 ± 0.0193	0.6971 ± 0.0203	0.7063 ± 0.1211	0.6762 ± 0.1801
JKNET	0.8349 ± 0.0184	0.8317 ± 0.0187	0.6816 ± 0.0193	0.6794 ± 0.0193	0.7600 ± 0.0301	0.7597 ± 0.0300
SGC	0.8698 ± 0.0381	0.8695 ± 0.0380	0.6748 ± 0.0162	0.6742 ± 0.0161	0.7755 ± 0.0158	0.7753 ± 0.0156
GraphSAGE	0.8444 ± 0.0278	0.8425 ± 0.0275	0.6864 ± 0.0237	0.6857 ± 0.0234	0.7666 ± 0.0191	0.7661 ± 0.0187

The bold values indicate that our proposed model achieves the best classification evaluation results.

As shown in Table 2, on the COPD dataset, DP-GA achieves the highest ACC of 88.73%, outperforming APPNP, DGI, GCN, JKNET, SGC, and GraphSAGE by 2.19%, 6.88%, 4.76%, 3.26%, 2.01%, and 5.08%, respectively. Similarly, in F1-score, it leads by margins of 2.21%, 6.97%, 5.88%, 6.67%, 2.04%, and 5.31% over the same baselines. This robust lead, particularly over strong feature-smoothing models like SGC (ACC: 86.98%), validates the advantage of our model's integrated structural and feature-based attention.

For the DM dataset, our model attains a top ACC of 72.10%, representing improvements of 5.04%, 4.89%, 3.28%, 5.78%, 6.85%, and 5.04% over the baselines. In F1-score, the corresponding improvements are 5.90%, 4.28%, 2.78%, 5.46%, 6.27%, and 4.49%. The significant gain over all models, highlights the effectiveness of the Bregman divergence module in capturing patient heterogeneity, which is crucial for modeling complex metabolic conditions like diabetes.

The most pronounced improvement is observed on the CHD dataset. Here, DP-GA's ACC of 79.76% exceeds that of the baselines by 5.70% (APPNP), 5.87% (DGI), 12.93% (GCN), 4.95% (JKNET), 2.85% (SGC), and 4.04% (GraphSAGE). The F1-score shows a similar trend. The exceptionally large margin over GCN (over 12%) underscores a critical finding: standard message-passing neural networks are prone to learning spurious correlations in clinical graphs. In contrast, DP-GA's disease potential-driven attention successfully constrains information flow to clinically plausible pathways, which is paramount for reliable prediction in cardiovascular etiology.

In summary, the systematic outperformance of DP-GA across all tasks and metrics confirms the efficacy of its core innovations: the fusion of structural position encoding, the dual-perspective modeling via Bregman divergence, and the incorporation of clinical logic. The model demonstrates not only higher accuracy but also greater robustness, as indicated by consistently lower standard deviations, establishing a new state-of-the-art for patient-centric comorbidity prediction.

### 4.4 Visualization results and analysis

**Figure 2:**
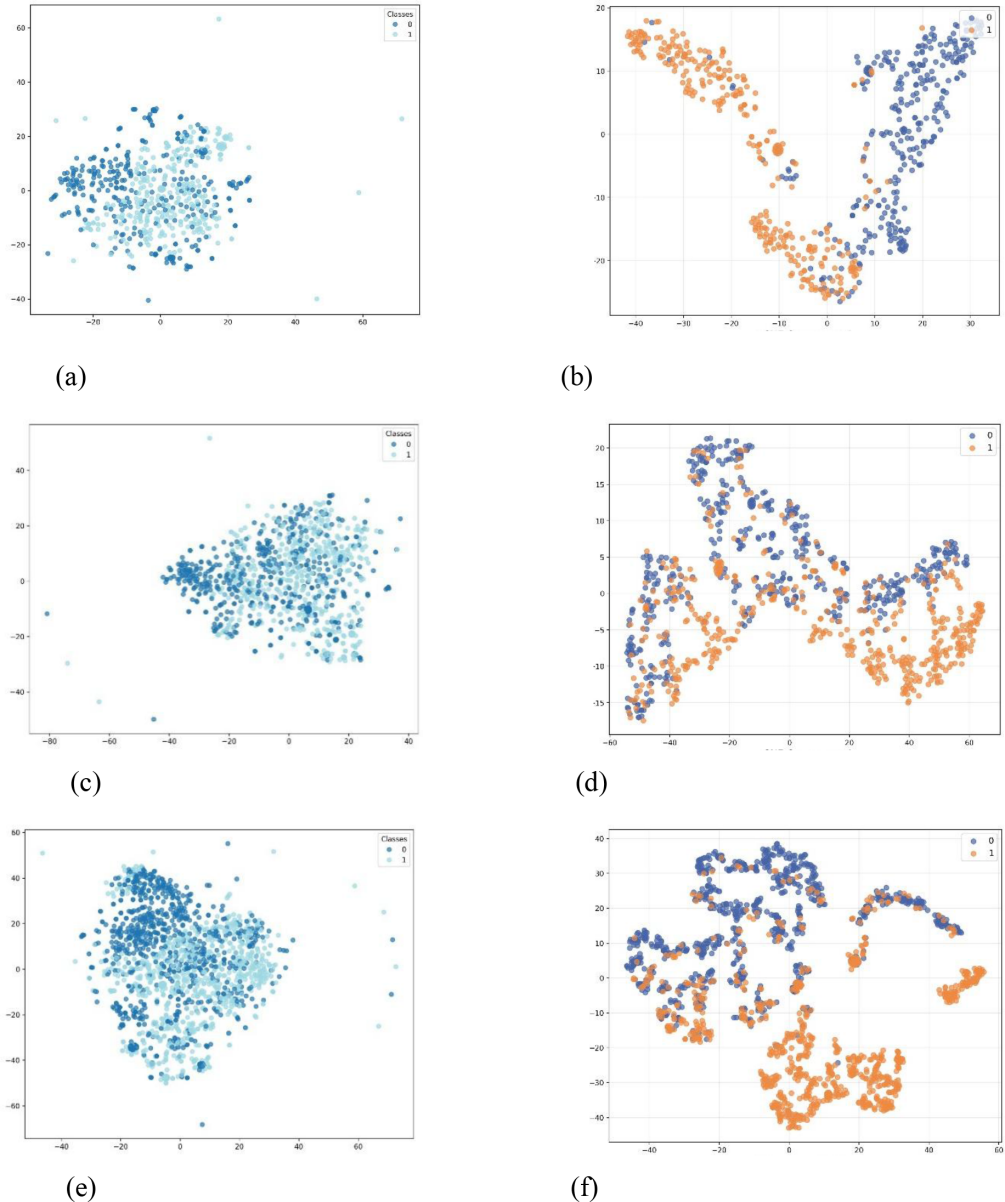
Visualizations of raw feature and DP-GA's embedding on each dataset. **(a)** Raw feature on COPD. **(b)** DP-GA's embedding on COPD. **(c)** Raw feature on DM. **(d)** DP-GA's embedding on MD. **(e)** Raw feature on CHD. **(f)** DP-GA's embedding on CHD.

To further validate the discriminative capability of the learned representations, we employed t-SNE to visualize the node embeddings in a two-dimensional space. Figure 2 presents a comparative visualization of the raw feature distributions against the embeddings generated by our DP-GA model.

Observations from the t-SNE plots reveal a significant qualitative improvement. In the visualization of the original high-dimensional features as depicted in Figures 2a, c, e, nodes belonging to the two target classes—patients with and without the target comorbidity—exhibit substantial overlap with no clear separation boundary. This indicates that the raw clinical features, while informative, are not readily separable for the downstream prediction task when viewed through a standard non-linear dimensionality reduction technique.

In contrast, the visualization of the embeddings produced by DP-GA depicted in Figures 2b, d, f, shows a markedly distinct pattern. The two patient cohorts form more compact and well-separated clusters in the latent space. This clear structural divergence between the classes provides direct visual evidence that our model successfully projects patients into an embedding space where the semantic information relevant to comorbidity risk is effectively encoded and amplified.

### 4.5 Findings of comorbidity network and pathological mechanism

**Figure 3:**
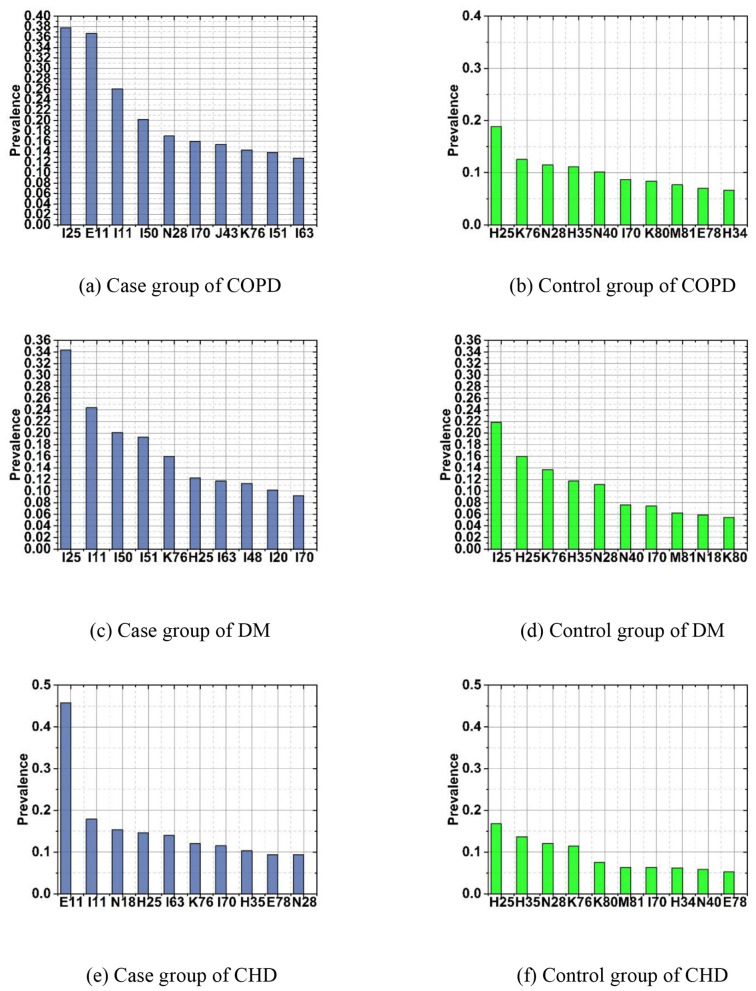
Epidemic rate of the case group and control group nodes of COPD, DM and CHD. **(a)** Case group of COPD. **(b)** Control group of COPD. **(c)** Case group of DM. **(d)** Control group of DM. **(e)** Case group of CHD. **(f)** Control group of CHD.

The above classification of comorbidities predicts which comorbidities a patient may develop in the future. However, clinical decision-making also requires an understanding of the mechanistic explanations for the reason of this patient is prone to these comorbidities. Analyzing the comorbidity patterns and exploring the pathological mechanisms can help identify the pathways between diseases, which is beneficial for identifying key risk factors to determine the priority intervention path, achieving intervention and etiological treatment. To this end, a case group of patients with hypertension combined with COPD, DM and CHD and a control group of patients with hypertension were included in the analysis model. This study included patients with primary hypertension as the control group and patients with primary hypertension the ending complicated with COPD, DM and CHD as the case group in Tables 3–5 and Figure 3.

**Table 3:** Description of the comorbidity network in the case and control group of COPD.

Case group							Control group						
^*^V1	V2	V1 degree	V2 degree	Phi	*t*-value	Number	^*^V1	V2	V1 degree	V2 degree	Phi	*t*-value	Number
C34	C77	16	7	0.5441	2.4265	6	E83	I97	8	8	0.7428	2.7179	6
I11	I25	49	71	0.2873	2.4915	30	E83	N18	8	18	0.6546	3.4637	8
I11	I50	49	38	0.3348	2.4358	21	H25	H35	54	32	0.3393	2.6010	18
I11	I51	49	26	0.3237	2.3458	16	H25	H40	54	11	0.2754	2.0657	8
I13	I51	7	26	0.4909	2.7603	7	H34	H35	19	32	0.5735	3.8348	15
I20	I25	18	71	0.3804	3.4169	17	I97	N18	8	18	0.6546	3.4637	8
I48	I50	18	38	0.4664	3.1639	14	K76	N28	36	33	0.4571	2.9966	18

^*^V denotes disease vertex.

As shown in Table 3 and Figures 3a, b, the case group has more disease nodes than the control group significantly, indicates that patients with COPD ending has multiple diseases, such as diabetes, coronary heart disease, heart failure, etc., which are closely related to COPD ending. This is consistent with the current literature (Lipton, 2018; Atkinson et al., 2023; Summers et al., 2024; Romano et al., 2023). However, if the control group has been controlled, reducing the complications of endocrine and cardiovascular diseases also helps to block the key nodes of disease progression to more serious diseases, thereby achieving the goal of preventing COPD and other more serious diseases.

As shown in Table 4 and Figures 3c, d, the case group has more disease nodes than the control group significantly, indicates that patients with DM ending has multiple diseases, such as coronary heart disease, heart failure, etc., which are closely related to the DM outcome. The disease nodes in the case group are more closely connected, and there are complex interactions among different disease nodes, especially secondary hypertension, heart failure, atrial fibrillation and flutter, complications of heart disease, etc. This is consistent with the current literature (Lipton, 2018; Atkinson et al., 2023; Summers et al., 2024; Romano et al., 2023; Wang et al., 2021; Cai, 2025; Yu et al., 2025; An et al., 2024; Recenti et al., 2021; Tang et al., 2022; Zhou et al., 2023).

**Table 4:** Description of the comorbidity network in the case and control group of DM.

Case group							Control group						
^*^V1	V2	V1 degree	V2 degree	Phi	*t*-value	Number	^*^V1	V2	V1 degree	V2 degree	Phi	*t*-value	Number
E21	N18	7	41	0.3990	2.7178	7	E83	N18	14	30	0.6211	4.1932	13
E83	N18	13	41	0.5471	4.0813	13	H25	H35	82	60	0.3542	3.3873	31
I11	I25	125	176	0.2588	3.5337	70	H34	H35	28	60	0.5266	4.7179	23
I11	I48	125	58	0.2559	2.9361	32	I20	I25	22	112	0.3771	4.2708	21
I11	I50	125	103	0.3840	4.6118	59	I97	N18	10	30	0.5657	3.6304	10
I11	I51	125	99	0.6197	8.7573	78	K76	N28	70	57	0.3291	2.8738	26

^*^V denotes disease vertex.

As shown in Table 5 and Figures 3e, f, we found type 2 diabetes, secondary hypertension and chronic kidney disease are the diseases with the highest prevalence in the case group. Early detection and prevention of these highly prevalent disease nodes can to a certain extent prevent patients from progressing to CHD (Wang et al., 2021; Cai, 2025; Yu et al., 2025; An et al., 2024; Recenti et al., 2021; Tang et al., 2022; Zhou et al., 2023).

**Table 5:** Hypertension comorbidity network in the case and control group of CHD.

Case group							Control group						
^*^V1	V2	V1 degree	V2 degree	Phi	*t*-value	Number	^*^V1	V2	V1 degree	V2 degree	Phi	*t*-value	Number
E11	H43	382	23	0.1391	2.7381	20	E83	N18	18	42	0.6072	4.8336	17
E11	I11	382	149	0.1429	2.8154	91	H25	H35	140	114	0.2602	3.1659	47
E11	N18	382	128	0.1761	3.4868	85	H25	H40	140	28	0.2547	3.0939	19
E21	N18	22	128	0.3243	3.8482	19	H25	H43	140	20	0.2232	2.6896	14
E83	I97	37	26	0.4305	2.8214	14	H34	H35	52	114	0.5181	6.4111	43
E83	N18	37	128	0.4737	6.0378	35	I97	N18	13	42	0.5464	4.1265	13
H25	H35	122	86	0.3729	4.4019	46	K76	N28	95	101	0.3759	4.0363	44

^*^V denotes disease vertex.

## 5 Conclusions

In this work, we proposed DP-GA, a novel graph neural network framework for predicting hypertension comorbidity risk. The core innovation lies in integrating a disease potential-driven attention mechanism with structured similarity-difference learning, enabling the model to capture the topological relationships within patient networks. This approach effectively addresses the challenge of early comorbidity risk screening by moving beyond correlation-based learning to model plausible pathogenic pathways.

Our experimental results on three major comorbidity prediction tasks (e.g., COPD, DM, and CHD) demonstrate that the proposed framework consistently and significantly outperforms existing graph learning baselines. However, the co-morbidity networks constructed based on the method of disease phenotype co-occurrence ignore the indirect associations between diseases, and can only capture the dominant co-morbidity patterns, which has certain limitations.

In our future work, there are still the following issues to be studied: (a) clustering patients within a inference-informed graph representation to uncover more precise comorbidity patterns and improve clustering accuracy; (b) developing a heterogeneous graph model that incorporates patient-specific attributes to better understand comorbidity distribution across subpopulations and support personalized healthcare; and (c) integrating multi-modal clinical data—such as laboratory results and imaging features—within a latent representation framework to enhance predictive performance and facilitate the discovery of novel biomarkers.

## Data Availability

The datasets presented in this article are not readily available because medical data is classified as private information and requires strict protection. It should be used in accordance with legal regulations. Requests to access the datasets should be directed to 203861@hospital.cqmu.edu.cn.
